# P-1678. Post Implementation Analysis of Inpatient Review by Infectious Diseases Pharmacists of Outpatient Parenteral Antimicrobial Therapy (OPAT) Prior to Discharge

**DOI:** 10.1093/ofid/ofae631.1844

**Published:** 2025-01-29

**Authors:** Anna Skillings, Delaney Hart, Emily Herstine, Krista Gens

**Affiliations:** Abbott Northwestern Hospital, part of Allina Health, Minneapolis, Minnesota; Abbott Northwestern Hospital, Eden Prairie, Minnesota; Abbott Northwestern Hospital, Eden Prairie, Minnesota; Abbott Northwestern Hospital, Eden Prairie, Minnesota

## Abstract

**Background:**

The Infectious Diseases Society of America OPAT guidelines recommend all patients should have ID expert review prior to initiation of OPAT. Prior to 2020, no formal OPAT program existed at this hospital system and prescribing was not limited to any specialty. However, in February 2020, the Outpatient Parenteral Antimicrobial Therapy - Inpatient Review (OPAIR) program was created in response to pre-implementation data showing only 16% of patients were discharged on appropriate therapy per IDSA OPAT guidelines.

Figure 1.

**Figure:**
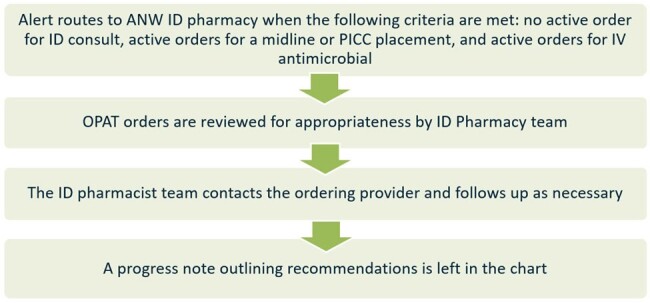

Outpatient Parenteral Antimicrobial Therapy Inpatient Review (OPAIR) program process

**Methods:**

This retrospective, observational chart review identified patients for data collection if they had pharmacist documentation of OPAIR review during their index hospital stay. Patients were included in the pre-implementation group if prescribed OPAT and admitted between January 1, 2017, and December 31, 2017, in the health system. Patients were included in the post-implementation group if 18 years and older, prescribed OPAT, and admitted between February 2, 2020, to August 1, 2023, at Abbott Northwestern, New Ulm, Owatonna, Faribault, or River Falls Hospitals. Exclusion criteria include OPAT prescribed by an Infectious Disease (ID) provider or prior to index hospitalization. Chi squared tests were performed for all outcomes.

Figure 2.

**Figure:**
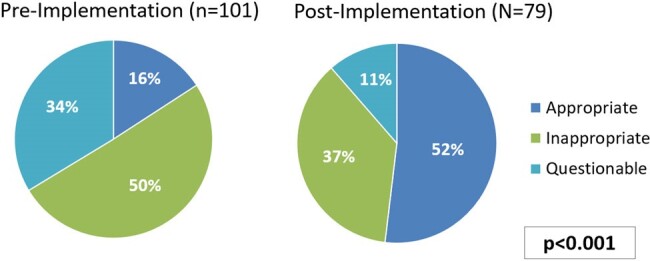

Primary outcome of OPAT appropriateness. Appropriateness defined as a composite outcome: appropriate monitoring or follow up​, indication​, antibiotic selection​, antibiotic dose/frequency/duration, and recommended laboratory monitoring.

**Results:**

There were 101 patients included in the pre and 79 in the post-implementation analysis. Internal Medicine was the most common prescriber in both the pre and post-implementation groups (60% vs 67%). OPAT appropriateness was significantly higher in the post-implementation group than in the pre-implementation group (52% (n=41/79) vs 16% (n=16/101), p< 0.001). The most common cause of inappropriate OPAT prescribing was a lack of monitoring or follow up (n=8/79). Complication rates were similar between pre and post groups (12% vs 18%, p=0.19) as was the 30-day readmission rate (17% vs 23%, p=0.32). Actual cost savings totaled $33,600 and maximum potential cost savings totaled to $214,200. The rates of midline utilization increased by 62% (p< 0.001).

Figure 3.

**Figure:**
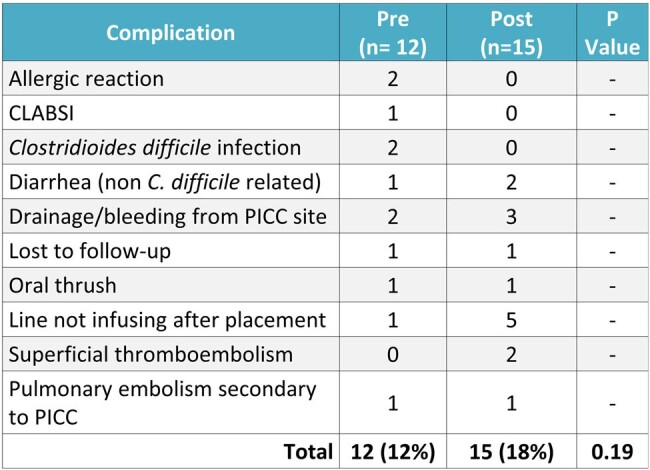

Secondary outcome of complication rate.

**Conclusion:**

The OPAIR program at this health system significantly increased non-ID provider OPAT prescribing appropriateness. These results support the creation of a new workflow and expansion of the OPAIR program.

Figure 4.

**Figure:**
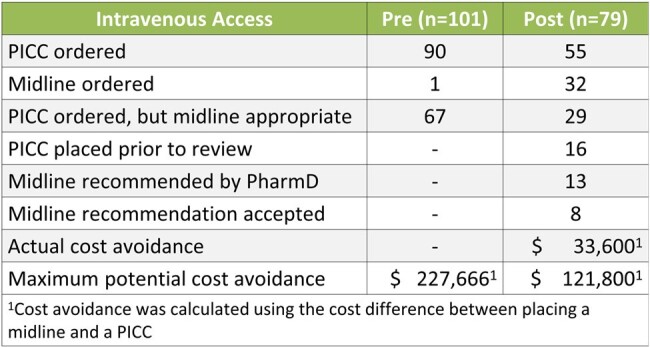

Secondary outcome of cost avoidance.

**Disclosures:**

**All Authors**: No reported disclosures

